# Belief in vaccine myths and vaccine uptake in Utah during the COVID-19 pandemic

**DOI:** 10.1016/j.pmedr.2023.102390

**Published:** 2023-09-01

**Authors:** Olusola A. Omisakin, Jessica D. Ulrich-Schad, Aaron Hunt, Jennifer E. Givens, Mitchell Beacham

**Affiliations:** a Department of Human Development and Family Studies, College of Health and Human Development, The Pennsylvania State University, 201 Henderson Building University Park, PA 16802, USA; b Department of Sociology & Anthropology, Utah State University, 0730 Old Main Hill, Logan, UT 84322-0730, USA; c Department of Kinesiology and Health Science, Utah State University, 7000 Old Main Hill, Logan, UT 84322-7000, USA

**Keywords:** Vaccine myths, COVID-19, Vaccine uptake, Beliefs, Utah

## Abstract

**Introduction:**

Since its emergence in December 2019, COVID-19 has caused millions of deaths worldwide. While vaccines are largely available in most places, including the United States (U.S.), vaccine uptake is lower than is desirable from a public health perspective.

**Objective:**

The objective of this paper is to examine belief in vaccine myths, including what individual and place characteristics underpin such beliefs, and uptake of vaccines – as well as the role of belief in myths in obtaining the COVID-19 vaccine.

**Methods:**

We use weighted survey data from an online panel of 529 Utah adult residents.

**Results:**

Sixty-nine percent of our sample was fully vaccinated at the time of taking the survey. We find that belief in vaccine myths varies among Utahns, with the highest percentage seeing the vaccines as unsafe because of their rapid development. Those who are older, are more religious (including members of The Church of Jesus Christ of Latter-day Saints), have less formal education, and are more conservative were more likely to have medium and/or higher levels of belief in vaccine myths. We find that belief in vaccine myths is associated with lower COVID-19 vaccine uptake, even when controlling for other factors.

**Conclusion:**

Understanding what drives vaccination uptake, including the role of belief in emerging vaccine myths, is important for public health measures in this and future outbreaks.

## Highlights

• COVID-19 vaccine uptake has been lower than desired from a public health perspective.
• One-third of survey respondents from Utah see the vaccine as unsafe because of rapid development and testing.
• Older, religious, less educated, and more conservative survey respondents had higher levels of belief in vaccine myths.
• Belief in vaccine myths is associated with lower COVID-19 vaccine uptake among Utah survey respondents.
• Understanding belief in myths is key for current and future public health measures given their spread on social media.

## 1 Introduction

COVID-19 was first detected in Wuhan, China in December of 2019. Over three and a half years later, almost seven million people have died from the virus worldwide (World Health Organization, 2023). In the United States (U.S.), over 6.2 million hospitalizations and 1.1 million deaths have been reported (Centers for Disease Control and Prevention, 2023). Vaccine development began in early 2020, with the first vaccine approved for emergency use in December 2020. Several COVID-19 vaccines were developed and released to the U.S. public in early 2021, first under Emergency Use Authorization by the U.S. Food and Drug Administration (FDA). Even during this period, vaccines were widely recommended by government agencies and medical professionals. The FDA granted full approval of the vaccines for people ages 16 and older starting in August 2021 (Food and Drug Administration, 2021).

Despite the widespread availability of vaccines and boosters in many parts of the world, uptake was not rapid enough to stop new strains from emerging. As of May 2023, about 80% of the U.S. population had an updated (bivalent) booster dose of the COVID-19 vaccine.^1^ Particular types of places and people have lower levels of COVID-19 vaccine uptake, leaving these populations more vulnerable. For instance, while there is variation between types of rural places, vaccine rates in rural counties have consistently lagged urban counties (Sun and Monnat, 2022, Dobis et al., 2021). Those with lower incomes and lower formal education levels, identifying with the Republican party, and who are non-white are also less likely to intend to or be vaccinated (Khubchandani et al., 2021, Malik et al., 2020, Yancy, 2020). While such studies document important contextual, demographic, and socioeconomic factors that play a role in vaccination rates, empirical studies documenting how attitudes, the spread of misinformation, and belief in myths are related to COVID-19 vaccine usage are still needed (Brennen et al., 2020, Loomba et al., 2021, Ullah et al., 2021).

Using data collected from an online panel of 635 residents of the U.S. state of Utah during the late summer of 2021, we seek to determine the extent of belief in COVID-19 vaccine myths among Utah adults and what factors are related to their beliefs. We define a vaccine myth as misinformation related to the coronavirus disease and COVID-19 vaccines. We also aim to determine what factors are related to COVID-19 vaccine uptake among Utah adults, particularly the role of belief in vaccine myths. Understanding what drives COVID-19 vaccine uptake, including the role of belief in vaccine myths, is important for public health measures in this and future outbreaks.

## 2 Methods

### 2.1 Data

An online panel of adults currently residing in the state of Utah was purchased from Qualtrics in 2021. From the 635 Utah residents who participated in the online survey, we excluded 10% with missing values on the key variables. As a result, the data in this manuscript are derived from the 529 Utahns who completed most of the survey.^2^ Respondents needed to be 18 years or older and live in Utah year-round or be a seasonal resident with current voter registration. The survey was conducted over a five-and-a-half-week span at the end of the summer of 2021, when there was a lull in positive cases, hospitalization rates, and deaths, and about 1.5 million people in the state were fully vaccinated (Utah Department of Health, 2022). The data used is not from a probability sample, meaning that the inferential statistics used should be interpreted with caution. Qualtrics provided some data that indicates the quality of the sample.^3^ We use iterative proportional fitting, or rake weights,^4^ by gender, age, education, party registration, and state region^5^ to create a more representative dataset of Utah adults. Even before weighting, the dataset was quite representative of the state on these variables apart from gender. This study was approved by the Utah State University Institutional Review Board (Protocol #12153).

### 2.2 Measures

**Fig. 1:**
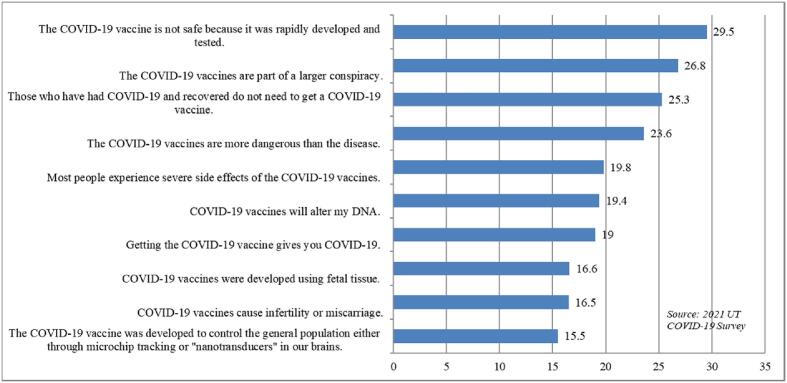
Percentage of respondents (Utah adults) who agreed or strongly agreed with statements about COVID-19 vaccine myths (n = 540).

Our first outcome variable, belief in vaccine myths, was created from 10 consecutive statements that measured respondents’ agreement about COVID-19 vaccines^6^ (see Fig. 1) The responses were coded using a Likert scale from strongly disagree (1) to strongly agree (5). However, we employed reverse coding to flip the questions that were asked in the opposite way, including statements four, five, and seven. A belief in vaccine myths index was calculated using principal component analysis of the 10 statements. Cronbach’s alpha for the 10 items was 0.89, indicating that the measures of belief in vaccine myths were internally consistent. The index score was then subdivided into tertiles (lowest, middle, and highest) to indicate the extent to which respondents believed in COVID-19 related myths. Our second dependent variable was vaccine uptake. Respondents were asked to indicate whether they were fully vaccinated (i.e., one or two doses depending on the vaccine; at the time of the survey this would have been considered fully vaccinated).

Sociodemographic factors were selected as independent variables, including gender, age, and religious affiliation. We collapsed 13 response options for current religion into three broad groups including no religious affiliation, Latter-day Saints (given their ubiquity in Utah),^7^ and other religions. We also included variables meant to represent socioeconomic status, i.e., education and social class. Race was categorized into two groups, white and others. Hispanic status was coded as ‘yes’ for respondents who self-identified as Hispanic, Latino, or Spanish origin, and ‘no’ if otherwise. We recoded political ideology in five categories as extremely liberal, fairly/somewhat liberal, don’t lean to either side, fairly/somewhat conservative, and extremely conservative. We included one measure of place effects given that existing research indicates that level of rurality of residence is important in COVID-19-related behavior (Ulrich-Schad et al., 2022). Level of rurality was assessed based on respondents’ perception about the place they currently live.^8^ Responses were classified into three categories to make comparisons easier, namely urban, rural, and in-between rural and urban (i.e., suburban, a mix).

### 2.3 Analysis

*First, w*e calculated descriptive statistics for the variables. For our analysis, we operationalized beliefs in vaccine myths as tertiles and used multinomial logistic regression. Operationalizing beliefs in vaccine myths as continuous index could have allowed us to estimate ordinary least squares (OLS) regression, but we found the distribution of the continuous index to be positively skewed and non-normal. Thus, the second step in our analysis involved estimating a multinomial logistic regression to determine the relationships between the sociodemographic factors and beliefs in vaccine myths. We estimated a multinomial logistic regression model of medium-level and high-level of beliefs in vaccine myths with low-level of belief as the common reference point.^9^ Finally, we estimated two binary logistic regression models to assess relationships between beliefs in vaccine myths and COVID-19 vaccine uptake, controlling for sociodemographic factors. We used rake weights in all analyses.

## 3 Results

### 3.1 Sample characteristics

Table 1 summarizes the respondents’ characteristics. After weighting, the proportions of women and men were similar (49.6% and 48.2% respectively), with 2.2% of adults in other gender categories. Respondents were, on average, 44 years old. Almost half of the respondents (49.8%) were members of the Latter-day Saints, followed by other religions (30.8%), while 19.4% had no religious affiliation. Most respondents had less than some college (31.4%) or some college education (38.9%), while one-fifth (20.4%) were college graduates and 9.3% had postgraduate education. The highest proportions of respondents belong to the middle and lower classes (35% and 34.9%, respectively) followed by 30.1% of respondents in the upper class. About 9 out of 10 identified themselves as non-Hispanic. Similarly, most of the sample (86.8%) was white. Respondents’ political ideology varied, where the lowest proportion (5.1%) thought of themselves as extremely liberal and the highest (33.7%) considered themselves as fairly/somewhat conservative. In terms of the rurality of residence, 29.8% lived in urban, 19% in rural, and just above one-half (51%) lived in-between rural and urban areas. Nearly one-third (28.9%) expressed the lowest level, 34.3% had a medium level, while 36.8% exhibited the highest level of belief in COVID-19 myths. At the time of the survey, more than two-thirds (68.9%) had been fully vaccinated, yet a considerable proportion was unvaccinated (31.1%).

**Table 1:** Weighted descriptive summary statistics of respondents’ sociodemographic characteristics, mean (SD) or % (survey conducted with Utah adults in 2021).

Variable	Variable Categories	Mean (SD) or %
Gender	Woman	49.6
	Man	48.2
	Other (e.g., transgender, gender non-conforming, prefer not to say)	2.2
Age	Mean (standard deviation)	43.5 (17.8)
Religion	No religious affiliation	19.4
	Latter-day Saints	49.8
	Other religion	30.8
Highest Level of Formal Education	High school or less	31.4
	Tech/some college	38.9
	College graduate	20.4
	Graduate school/professional degree	9.3
Social Class	Upper class	30.1
	Middle class	35
	Lower class	34.9
Race	White	86.8
	Non-white	13.2
Hispanic Status	Non-Hispanic	89
	Hispanic	11
Political Ideology	Extremely liberal	5.1
	Fairly/somewhat liberal	17.5
	Don't lean to either side	29
	Fairly/somewhat conservative	33.7
	Extremely conservative	14.7
Level of Rurality	Urban	29.8
	In-between rural and urban	51.2
	Rural	19
Level of Belief in Vaccine Myths	Lowest	28.9
	Medium	34.3
	Highest	36.8
Vaccine Uptake	No	31.1
	Yes	68.9
Weighted Sample (N)		**529**

### 3.2 Belief in vaccine myths

In Fig. 1, we present the level of belief in COVID-19 vaccine myths. Among Utah adults, belief was highest for the rapid production of COVID-19 vaccines (i.e., nearly 30% of adults agreed or strongly agreed that COVID-19 vaccines are not safe because they were rapidly developed and tested). The second most believed myth was related to the issue of conspiracy (26.8%), followed by the belief that people who naturally recovered after getting COVID-19 do not need vaccination (25.3%). Utahns were least likely to believe that the vaccines were developed to control the population (15.5%), cause infertility or miscarriage (16.5%), or that they were developed using fetal tissue (16.6%).

### 3.3 Factors related to belief in vaccine myths

Table 2 provides the results of multinomial logistic regression showing the relationship between sociodemographic variables and the relative risk ratios of beliefs in vaccine myths among Utah adults. Respondents’ age, religious affiliation, level of education, and political ideology statistically influenced the levels of belief in vaccine myths. There were not significant associations of other variables with levels of belief in vaccine myths. For each additional year of age, the relative risk of exhibiting the highest level of belief in vaccine myths, instead of the lowest level, decreased by 4%.

**Table 2:** Multinomial logistic regression of factors that contribute to vaccine myths among respondents with ‘lowest-level myth’ as the base outcome (survey conducted with Utah adults in 2021).

Variables	Variable Categories	Medium-level Myth RRR (SE)	Highest-level Myth RRR (SE)
Gender	Woman	(ref)	(ref)
	Man	0.891 (0.278)	1.315 (0.419)
	Other (e.g., transgender, gender non-conforming, prefer not to say)	1.480 (0.884)	0.449 (0.405)
Age	N/A	0.984 (0.010)	0.963 (0.010) ***
Religion	No religious affiliation	(ref)	(ref)
	Latter-day Saints	2.680 (0.975) **	1.053 (0.412)
	Other religion	4.082 (1.629) **	2.564 (1.058) *
Highest Level of Formal Education	High school or less	(ref)	(ref)
	Tech/some college	0.435 (0.181)*	0.618 (0.271)
	College graduate	0.300 (0.141) *	0.273 (0.142) *
	Graduate school/professional degree	0.229 (0.135) *	0.157 (0.107) **
Social Class	Upper class	(ref)	(ref)
	Middle class	1.538 (0.527)	1.387 (0.502)
	Lower class	1.286 (0.484)	1.896 (0.726)
Race	White	(ref)	(ref)
	Non-white	1.238 (0.568)	1.825 (0.849)
Hispanic Status	Non-Hispanic	(ref)	(ref)
	Hispanic	2.762 (1.615)	2.713 (1.568)
Political Ideology	Extremely liberal	(ref)	(ref)
	Fairly/somewhat liberal	0.672 (0.332)	0.914 (0.583)
	Don't lean to either side	2.135 (0.993)	3.447 (2.090) *
	Fairly/somewhat conservative	2.013 (1.018)	6.167 (3.966) **
	Extremely conservative	3.331 (1.842) *	9.951 (7.036) **
Level of Rurality	Urban	(ref)	(ref)
	In-between rural and urban	1.063 (0.333)	0.873 (0.288)
	Rural	1.374 (0.553)	1.621 (0.661)
Constant		0.911 (0.642)	1.435 (1.162)

* p-value < 0.05, ** p-value < 0.01, *** p-value < 0.001; RRR Relative risk ratio.

Religious affiliation was associated with vaccine myth belief. The relative risk of exhibiting medium level of belief in vaccine myths, instead of the lowest level, increased by 2.7 times among adults who were members of the Latter-day Saints and about 4.1 times among those who practiced other religions, as compared to adults who had no religious affiliation. Similarly, the relative risk of exhibiting the highest level of belief in vaccine myths, instead of the lowest level, was 2.6 times higher for adults who practiced other religions than adults who had no religious affiliation.

Additionally, beliefs in vaccine myths were less likely among adults who attained college degree and postgraduate education as opposed to adults who had less than some college. For instance, the relative risk of displaying the highest level of belief in vaccine myths, instead of the lowest level, decreased by 63% for adults who attained a college degree and decreased by 84% for adults who attained postgraduate education, compared to adults who had less than some college, suggesting an inverse relationship between level of education and beliefs in vaccine myths.

Results indicate that adults who were extremely conservative were 3.3 times more likely to express the medium level of belief in vaccine myths than adults who were extremely liberal. In addition, the relative risk of expressing the highest level of belief in vaccine myths, instead of the lowest level, was 6.2 times higher for adults who were fairly conservative and 10 times higher for adults who were extremely conservative, as compared to those who were extremely liberal. We conducted a sensitivity analysis using the continuous vaccine myth index and OLS regression to increase confidence that our results are not biased because of the choice of operationalization of beliefs in vaccine myth. The conclusions were similar when compared to our analysis using the multinomial logistic regression.

### 3.4 Belief in vaccine myths and vaccine uptake

To examine predictors of COVID-19 vaccine uptake among Utah adults, we estimated two binary logistic regression models shown in Table 3. The first model shows a significant relationship between sociodemographic variables such as age, social class, and political ideology and the odds ratios of vaccine uptake. For each additional year of age, the odds of COVID-19 vaccine uptake slightly increased by 4%, suggesting that older adults were more likely to be vaccinated than younger adults. The odds of COVID-19 vaccine uptake were 63% less among adults who belonged to the lower category of social class than their counterparts who belonged to the upper category. In terms of political ideology, the odds of COVID-19 vaccine uptake were 81% less among adults who were fairly conservative and 77% less among adults who were extremely conservative relative to those who were extremely liberal.

**Table 3:** Binary logistic regression showing the predictors of vaccine uptake among respondents (survey conducted with Utah adults in 2021).

Variables	Variable Categories	Vaccine Uptake OR (SE)	Vaccine Uptake AOR (SE)
Level of Belief in Vaccine Myths	Lowest		(ref)
	Medium		0.075 (0.050) ***
	Highest		0.006 (0.004) ***
Gender	Woman	(ref)	(ref)
	Man	1.222 (0.350)	1.757 (0.604)
	Other (e.g., transgender, gender non-conforming, prefer not to say)	3.577 (3.073)	3.420 (2.081) *
Age	N/A	1.039 (0.008) ***	1.033 (0.011) **
Religion	No religious affiliation	(ref)	(ref)
	Latter-day Saints	1.788 (0.679)	1.868 (0.855)
	Other religion	1.023 (0.383)	1.383 (0.670)
Highest Level of Formal Education	High school or less	(ref)	(ref)
	Tech/some college	0.798 (0.241)	0.775 (0.288)
	College graduate	1.747 (0.736)	1.223 (0.626)
	Graduate school/professional degree	2.824 (1.888)	1.518 (1.150)
Social Class	Upper class	(ref)	(ref)
	Middle class	0.856 (0.309)	0.942 (0.440)
	Lower class	0.368 (0.127) **	0.381 (0.163) *
Race	White	(ref)	(ref)
	Non-white	0.624 (0.242)	0.670 (0.273)
Hispanic Status	Non-Hispanic	(ref)	(ref)
	Hispanic	2.313 (1.018)	4.570 (2.306) **
Political Ideology	Extremely liberal	(ref)	(ref)
	Fairly/somewhat liberal	0.520 (0.301)	0.440 (0.283)
	Don't lean to either side	0.303 (0.158) *	0.488 (0.246)
	Fairly/somewhat conservative	0.185 (0.100) **	0.392 (0.206)
	Extremely conservative	0.233 (0.147) *	0.722 (0.461)
Level of Rurality	Urban	(ref)	(ref)
	In-between rural and urban	0.775 (0.232)	0.540 (0.198)
	Rural	0.551 (0.222)	0.521 (0.254)
Constant		1.926 (1.361)	43.034 (40.713) ***

* p-value < 0.05, ** p-value < 0.01, *** p-value < 0.001; OR Odds ratio; AOR Adjusted Odds ratio.

The second model shows the relationship between levels of belief in vaccine myths and the odds of COVID-19 vaccine uptake, controlling for sociodemographic and place measures. The likelihood of being vaccinated is significantly reduced if respondents believe in vaccine myths (i.e., the odds of being fully vaccinated was lower by 92% for adults who expressed a medium level and lower by 99.9% for adults who expressed the highest level, compared to those who expressed lowest level of belief in vaccine myths). The association of vaccine uptake with age and social class remained significant. Additionally, those in the “other” gender category and those with Hispanic ethnicity had higher odds of being vaccinated. In the second model, the results show that political ideology was not associated with the odds of vaccine uptake, perhaps because there was such a strong relationship between political ideology and belief in vaccine myths. In the two binary logistic regression models, we were not able to detect any association between vaccine uptake and religion, education, race, or rurality of residence.

## 4 Discussion

Despite their benefits, not everyone who is eligible to receive a vaccine will follow recommendations, as has been the case for COVID-19 vaccines. In our study, about 69% of respondents were fully vaccinated from COVID-19 during the summer of 2021, which was slightly higher than the national and state average at the time (Utah Department of Health, 2022). Vaccine hesitancy or refusal is not a new social phenomenon in the U.S. or elsewhere (Dubé et al., 2015). Research shows many factors can contribute to uptake for both vaccines in general, and COVID-19 specifically, and these factors can broadly be categorized into individual/group level factors, contextual determinants, or issues specifically related to vaccine types (MacDonald, 2015). Additionally, people with negative attitudes towards vaccinations are less likely to receive the COVID-19 vaccine than those with positive attitudes (Khairat et al., 2022). Higher chances of getting the COVID-19 vaccination are related to sociodemographic factors, such as older age and higher educational attainment (Khairat et al., 2022, Galanis et al., 2022). The internet and social media have played a key role in helping anti-vaccination activists spread misinformation, including during the COVID-19 pandemic (Burki, 2020). Research also documented inaccuracies in perceptions about the COVID-19 pandemic, including false optimism, or unrealistic expectations about the course of the pandemic and belief in myths (e.g., “Vaccinations for COVID-19 implant microchips to track people”) (Ulrich-Schad et al., 2022, Hamilton, 2022).

In this paper we sought to understand belief in COVID-19 vaccine myths and how it played a role in vaccine uptake given indications that it was important to examine the influence of these beliefs in more depth. Those who are older, more religious (including Latter-day Saints church members), have less formal education, and are more conservative, are more likely to have medium and/or higher levels of belief in vaccine myths. Our finding with regards to age suggests that younger adults had greater likelihood of having beliefs in vaccine myths, compared to older, more mature adults who are less susceptible to misinformation (Auxier and Anderson, 2021, Roozenbeek et al., 2020). Younger adults are also more likely to be on social media (Freeman et al., 2022) which is where many anti-vaccine messages have been spread (Burki, 2020). Our findings with regards to religion align with other emerging research about the links between religiosity and belief in COVID-19 conspiracies (Freeman et al., 2022). If supportive of such efforts, religious organizations and leaders may then be a good avenue for public health responses in combating pandemic-related myths and similarly encouraging their members to get vaccinated. In Utah, there is some evidence that after a statement on August 12, 2021, in which Latter-day Saints leaders urged members to get their shots, vaccine rates which had been stagnating went up (Alberty, 2021).

Our research shows that those with less education are more likely to believe in COVID-19 myths, which also aligns with Freeman et al. (Freeman et al., 2022). Existing research indicates that those who are more educated also have a greater ability to evaluate scientific information (Crowell and Schunn, 2016), which may be what we saw in this case where new information was rapidly becoming available. Finally, there is a large and growing body of research indicating the politicization of the pandemic including a positive relationship between political conservatism and belief in inaccurate information about COVID-19. For instance, while not identical to vaccine myths, some research looked at false optimism during the first summer of the pandemic (2020) and found that Republicans were much more likely to express this belief (Hamilton and Safford, 2021). Hamilton (Hamilton, 2022) also found that those who support former President Trump were more likely to believe that the COVID-19 vaccinations implant a microchip. Overall, our findings with regards to the factors that are related to belief in vaccine myths are in line with existing studies.

Limited research to date has attempted to understand how belief in vaccine myths is related to COVID-19 vaccination as we do here. We find that belief in vaccine myths is associated with lower likelihood of COVID-19 vaccine uptake, even when controlling for other factors. The results indicated that odds of being fully vaccinated were highly associated with beliefs in vaccine myths. The association of vaccine uptake with age and social class remained significant in our full model. Older people tend to have a higher risk and feel more susceptible to adverse outcomes (Chia and Hartanto, 2021), despite belief in myths in this case, which may explain this finding. Those in higher social classes have many reduced barriers to obtaining vaccines, including access and the ability to take off work if sick from vaccination. Lower levels of vaccine hesitancy among higher class individuals are consistent with existing studies (Khubchandani et al., 2021). Additionally, we found a relationship between those who do not identify as women or men and those who are Hispanic and vaccine uptake, but our sample size for these groups was small so the true impact may differ and we interpret these results with caution. The finding that the relationship between political ideology and vaccine uptake disappears with the inclusion of belief in vaccine myths in our model further solidifies the degree to which conservatism and belief in vaccine myths are intertwined.

The rapid dissemination of vast amounts of information related to the COVID-19 pandemic has been described by the WHO and other global organizations as an “infodemic”, which made it difficult for the public to critically evaluate information, especially coupled with the rapidly evolving science (WHO et al., 2019). Throughout the pandemic, many people were exposed to misinformation about COVID-19 vaccines which research has shown reduces intent to be vaccinated (Loomba et al., 2021). Our research further reinforces this finding regarding the association between belief in vaccine myths and lower vaccine uptake using data from the state of Utah.

This study has some important limitations that should be reiterated. First, while this study provides some novel insights, the data is from a nonprobability sample and is focused only on adult vaccine uptake in one state. Second, while this is not within the scope of this paper, other research indicates that trust in information from different sources about COVID-19 is important to consider in understanding vaccine uptake (Chia and Hartanto, 2021) and should be investigated in future studies.

## 5 Conclusions

Understanding factors related to vaccine uptake will help improve public health responses to outbreaks in the future. While the COVID-19 pandemic has passed its peak, new variants continue to emerge and new viruses will surely arise. Our findings suggest that paying attention to myths related to the pandemic and the factors related to belief in them will be important in implementing effective public health strategies in the future. In addition, the relationship between conservative political ideology, belief in vaccine myths, and vaccine uptake is one that must be paid attention to. We see these findings as being particularly relevant to researchers and public health professionals working in similar politically conservative and religious contexts.

## Funding source

Funding for collecting the survey data was provided by a Utah State University College of Humanities and Social Sciences Creative Activity and Research Enhancement Grant. Publication of this research was also supported by the Utah Agricultural Experiment Station (UAES), Department of Kineseology and Health Science, Library, and College of Humanities and Social Sciences at Utah State University, and approved as UAES journal paper number 9712.

## CRediT authorship contribution statement

**Olusola A. Omisakin:** Conceptualization, Methodology, Formal analysis, Writing – original draft. **Jessica D. Ulrich-Schad:** Conceptualization, Methodology, Writing – original draft, Supervision, Project administration, Funding acquisition. **Aaron Hunt:** Writing – original draft. **Jennifer E. Givens:** Methodology, Formal analysis, Writing – original draft, Funding acquisition. **Mitchell Beacham:** Conceptualization, Methodology, Writing – original draft.

## Declaration of Competing Interest

The authors declare that they have no known competing financial interests or personal relationships that could have appeared to influence the work reported in this paper.

## Data Availability

Data will be made available on request.

## Footnotes

1 See the Centers for Disease Control: https://covid.cdc.gov/covid-data-tracker/#vaccinations_vacc-people-booster-percent-pop5.

2 The analytic (unweighted) sample is 565, but when the weights are applied in the analysis, the weighted total is 529.

3 Data provided from Qualtrics included: attrition rate = 42% (1-good completes (partial completes + good completes); response rate = 73% (good completes/all people who entered the survey); and engagement rate = 52% (good completes/all the people who saw the survey posted online). Qualtrics considers a “good complete” to be a “respondent that successfully passes all screening questions in a survey, meets quotas in a survey, makes it to the end of the survey without speeding, and is not removed for a quality check such as straight-lining, gibberish responses, or contradictory responses.”.

4 Rake weights use variables for which the population distribution is known to iteratively adjust the weight for each case until the sample distribution matches with the population on the specified variables. This method has been shown to perform well in comparison to more elaborate weighting options (Mercer et al., 2018).

5 Data from the 2019 American Community Survey (ACS) is used to construct the weights for gender, age, and education; 2019 data from the State of Utah (https://voteinfo.utah.gov/current-voter-registration-statistics) is used for the party registration weight; and the regions outlined by the Utah Department of Health and Human Services (https://hs.utah.gov/soc-contact) are used to construct the region weight.

6 The questions on vaccine myths were derived by the authors from an article published online in December 2020 by the Mayo Clinic with common myths at the time: https://newsnetwork.mayoclinic.org/discussion/covid-19-vaccine-myths-debunked/.

7 The full name is The Church of Jesus Christ of Latter-day Saints, which we also refer to in the paper as Latter-day Saints.

8 Like Ulrich-Schad et al. (2022), we use a subjective measure of rurality of residence. Given the large size of many Utah counties, this provides better alignment with residents’ experiences, and there is strong alignment between the subjective measures and USDA county-level typologies.

9 Considering that our measure of beliefs in vaccine myths has three ordered categories, the proportional odds model may be used in this situation. But the key assumption for this model was not fully met when examined using the Brant test. Our research objectives may also be addressed without interpretation about ordering of beliefs in vaccine myths. In any case, multinomial logistic regression can be utilized for unordered and ordered categories, such as when the proportional odds assumption does not seem agreeable.
