# Supplementary material for: Belief in vaccine myths and vaccine uptake in Utah during the COVID-19 pandemic
Source: Prev Med Rep. 2023 Sep 1;36:102390. doi: 10.1016/j.pmedr.2023.102390 (PMC10500446; doi:10.1016/j.pmedr.2023.102390)
Supplement: Supplementary data 1 [file mmc1.docx]

Appendices

I. Histogram showing the distribution of scores for the vaccine myth index created from Utahn adults’ agreement with statements about COVID-19 vaccines.


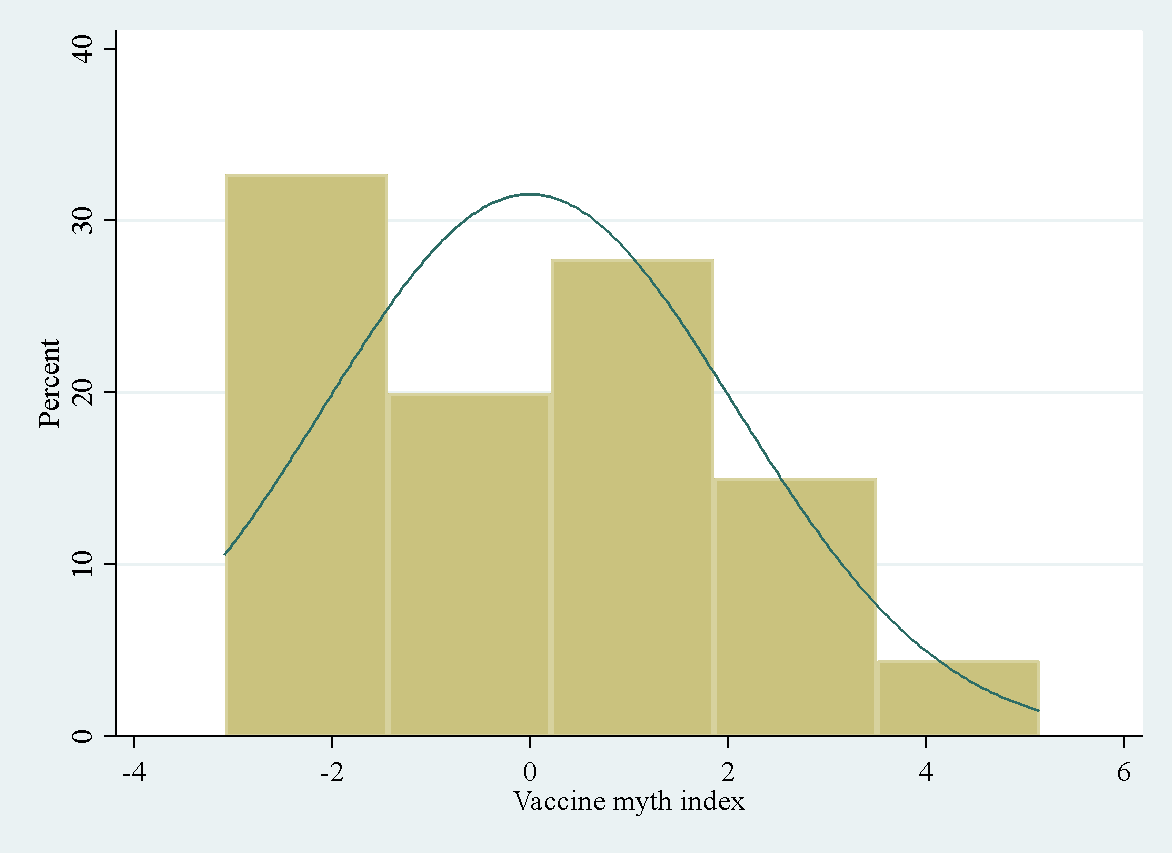


II. Descriptive summary statistics of vaccine myth index created from Utahn adults’ agreement with statements about COVID-19 vaccines

|  | Percentiles | Smallest |  |  |  |
| --- | --- | --- | --- | --- | --- |
| 1% | -3.092 | -3.092 |  |  |  |
| 5% | -2.943 | -3.092 |  |  |  |
| 10% | -2.713 | -3.092 |  | Obs | 565 |
| 25% | -1.933 | -3.092 |  | Sum of Wgt. | 565 |
|  |  |  |  |  |  |
| 50% | -0.011 |  |  | Mean | -0.007 |
|  |  | Largest |  | Std. Dev. | 2.083 |
| 75% | 1.538 | 5.028 |  |  |  |
| 90% | 2.862 | 5.032 |  | Variance | 4.340 |
| 95% | 3.375 | 5.119 |  | Skewness | 0.223 |
| 99% | 4.802 | 5.144 |  | Kurtosis | 2.051 |

III. Skewness and kurtosis tests for normality of vaccine myth index created from Utahn adults’ agreement with statements about COVID-19 vaccines

|  |  |  | **Joint test** | |
| --- | --- | --- | --- | --- |
| **Variable** | **Pr(skewness)** | **Pr(kurtosis)** | **Adj chi2(2)** | **Prob>chi2** |
| **Myth index** | 0.0304 | 0.0000 | 72.93 | 0.0000 |

IV. Coefficients in OLS regression model of vaccine myth index on socio-demographic characteristics of Utahn adults

| **Variables** | **Categories** | **Beta (SE)** | **[95% CI]** |
| --- | --- | --- | --- |
| **Gender** | Woman | (ref) |  |
|  | Man | 0.333 (0.222) | [-0.103, 0.770] |
|  | Others | -0.715 (0.430) | [-1.561, 0.130] |
| **Age** | N/A | **-0.029 (0.006) ***** | **[-0.042, -0.017]** |
| **Religion** | No religious affiliation | (ref) |  |
|  | Latter-Day Saints (LDS) | 0.115 (0.266) | [-0.407, 0.636] |
|  | Other religion | **0.642 (0.272) *** | **[0.107, 1.178]** |
| **Highest level of education** | Less than some college | (ref) |  |
|  | Some college | -0.317 (0.272) | [-0.851, 0.217] |
|  | College graduate | **-0.914 (0.328) **** | **[-1.558, -0.270]** |
|  | Postgraduate | **-1.372 (0.371) ***** | **[-2.101, -0.643]** |
| **Level of rurality** | Urban | (ref) |  |
|  | Rural | 0.432 (0.305) | [-0.167, 1.032] |
|  | In-between rural and urban | 0.035 (0.214) | [-0.384, 0.455] |
| **Social class** | Upper class | (ref) |  |
|  | Middle class | -0.004 (0.242) | [-0.479, 0.471] |
|  | Lower class | 0.374 (0.274) | [-0.165, 0.912] |
| **Race** | White | (ref) |  |
|  | Others | **0.639 (0.320) *** | **[0.011, 1.267]** |
| **Hispanic status** | Non-Hispanic | (ref) |  |
|  | Hispanic | 0.209 (0.321) | [-0.423, 0.841] |
| **Political ideology** | Extremely liberal | (ref) |  |
|  | Fairly/somewhat liberal | 0.106 (0.418) | [-0.716, 0.928] |
|  | Don't lean to either side | **0.835 (0.404) *** | **[0.042, 1.628]** |
|  | Fairly/somewhat conservative | **1.392 (0.419) **** | **[0.569, 2.215]** |
|  | Extremely conservative | **1.841 (0.486) ***** | **[0.887, 2.796]** |
| Constant |  | 0.205 (0.517) | [-0.810, 1.220] |

** p-value < .05, ** p-value < .01, *** p-value < .001; CI: Confidence Intervals*

V. Binary logistic regression showing the predictors of vaccine uptake among Utahns.

| **Variables** | **Categories** | **Vaccine uptake OR (SE)** | **[95% CI]** |
| --- | --- | --- | --- |
| **Vaccine myth index** | N/A | **0.345 (0.041) ***** | **[0.274, 0.435]** |
| **Gender** | Woman | (ref) |  |
|  | Man | **2.016 (0.719) *** | **[1.002, 4.057]** |
|  | Others | 3.226 (2.140) | [0.879, 11.839] |
| **Age** | N/A | **1.030 (0.011) **** | **[1.009, 1.051]** |
| **Religion** | No religious affiliation | (ref) |  |
|  | Latter-Day Saints (LDS) | **2.472 (1.139) *** | **[1.002, 6.098]** |
|  | Other religion | 1.695 (0.820) | [0.657, 4.372] |
| **Highest level of education** | Less than some college | (ref) |  |
|  | Some college | 0.574 (0.223) | [0.268, 1.228] |
|  | College graduate | 1.030 (0.571) | [0.348, 3.050] |
|  | Postgraduate | 0.888 (0.750) | [0.170, 4.647] |
| **Level of rurality** | Urban | (ref) |  |
|  | Rural | 0.619 (0.295) | [0.244, 1.575] |
|  | In-between rural and urban | 0.730 (0.287) | [0.338, 1.576] |
| **Social class** | Upper class | (ref) |  |
|  | Middle class | 0.722 (0.345) | [0.283, 1.844] |
|  | Lower class | **0.326 (0.146) *** | **[0.136, 0.782]** |
| **Race** | White | (ref) |  |
|  | Others | 0.829 (0.376) | [0.341, 2.015] |
| **Hispanic status** | Non-Hispanic | (ref) |  |
|  | Hispanic | **4.431 (2.274) **** | **[1.621, 12.117]** |
| **Political ideology** | Extremely liberal | (ref) |  |
|  | Fairly/somewhat liberal | 0.513 (0.358) | [0.131, 2.012] |
|  | Don't lean to either side | 0.414 (0.254) | [0.125, 1.376] |
|  | Fairly/somewhat conservative | 0.374 (0.232) | [0.111, 1.264] |
|  | Extremely conservative | 0.969 (0.793) | [0.195, 4.818] |
| Constant |  | 3.267 (2.773) | [0.619, 17.242] |

** p-value < .05, ** p-value < .01, *** p-value < .001; CI: Confidence Intervals*

VI. Mean predicted probabilities of vaccine uptake across levels of belief in vaccine myth among Utahn adults


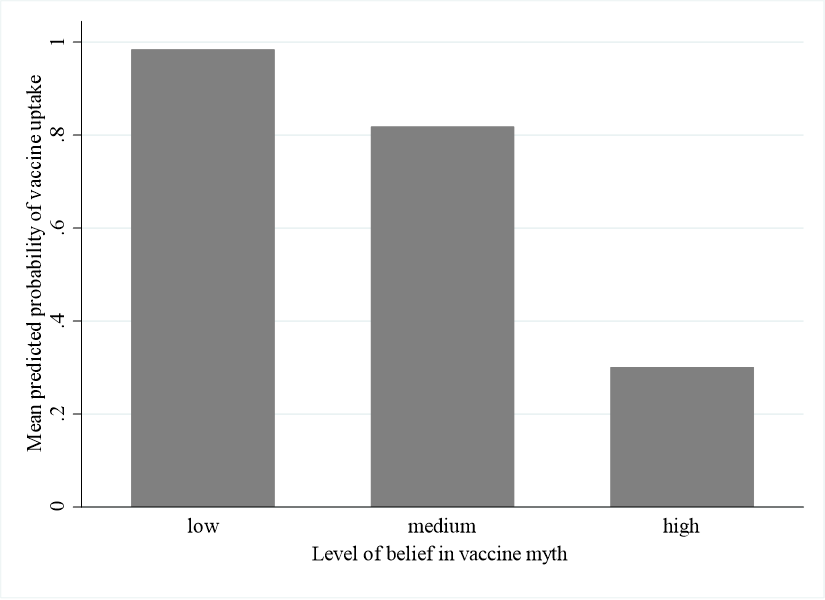


VII. Multinomial logistic regression of the association between gender and vaccine myths among Utahn adults with ‘lowest-level myth’ as the base outcome

| **Variables** | **Categories** | **Medium-level myth RRR (SE)** | **Highest-level myth RRR (SE)** |
| --- | --- | --- | --- |
| **Gender** | Woman | (ref) | (ref) |
|  | Man | 0.648 (0.161) | 0.744 (0.183) |
|  | Others | 1.234 (0.710) | 0.379 (0.363) |
| Constant |  | 1.460 (0.222) * | 1.506 (0.226) ** |

** p-value < .05, ** p-value < .01, *** p-value < .001; RRR* Relative risk ratio

VIII. Multinomial logistic regression of the association between age in years and vaccine myths among Utahn adults with ‘lowest-level myth’ as the base outcome

| **Variables** | **Medium-level myth RRR (SE)** | **Highest-level myth RRR (SE)** |
| --- | --- | --- |
| **Age** | 0.985 (0.007) * | 0.972 (0.007) *** |
| Constant | 2.333 (0.817) * | 4.467 (1.525) *** |

** p-value < .05, ** p-value < .01, *** p-value < .001; RRR* Relative risk ratio

IX. Multinomial logistic regression of the association between religious affiliation and vaccine myths among Utahn adults with ‘lowest-level myth’ as the base outcome

| **Variables** | **Categories** | **Medium-level myth RRR (SE)** | **Highest-level myth RRR (SE)** |
| --- | --- | --- | --- |
| **Religion** | No religious affiliation | (ref) | (ref) |
|  | Latter-Day Saints (LDS) | 2.072 (0.687) * | 1.008 (0.312) |
|  | Other religion | 2.883 (1.035) ** | 2.068 (0.687) * |
| Constant |  | 0.601 (0.169) | 1.030 (0.254) |

** p-value < .05, ** p-value < .01, *** p-value < .001; RRR* Relative risk ratio

X. Multinomial logistic regression of the association between highest level of education and vaccine myths among Utahn adults with ‘lowest-level myth’ as the base outcome

| **Variables** | **Categories** | **Medium-level myth RRR (SE)** | **Highest-level myth RRR (SE)** |
| --- | --- | --- | --- |
| **Highest level of education** | Less than some college | (ref) | (ref) |
|  | Some college | 0.454 (0.165) * | 0.604 (0.217) |
|  | College graduate | 0.266 (0.101) ** | 0.213 (0.085) *** |
|  | Postgraduate | 0.160 (0.077) *** | 0.087 (0.048) *** |
| Constant |  | 2.754 (0.834) ** | 2.820 (0.852) ** |

** p-value < .05, ** p-value < .01, *** p-value < .001; RRR* Relative risk ratio

XI. Multinomial logistic regression of the association between level of rurality and vaccine myths among Utahn adults with ‘lowest-level myth’ as the base outcome

| **Variables** | **Categories** | **Medium-level myth RRR (SE)** | **Highest-level myth RRR (SE)** |
| --- | --- | --- | --- |
| **Level of rurality** | Urban | (ref) | (ref) |
|  | Rural | 1.200 (0.439) | 1.219 (0.435) |
|  | In-between rural and urban | 0.872 (0.247) | 0.681 (0.192) |
| Constant |  | 1.240 (0.284) | 1.499 (0.330) |

** p-value < .05, ** p-value < .01, *** p-value < .001; RRR* Relative risk ratio

XII. Multinomial logistic regression of the association between social class and vaccine myths among Utahn adults with ‘lowest-level myth’ as the base outcome

| **Variables** | **Categories** | **Medium-level myth RRR (SE)** | **Highest-level myth RRR (SE)** |
| --- | --- | --- | --- |
| **Social class** | Upper class | (ref) | (ref) |
|  | Middle class | 1.830 (0.537) * | 1.568 (0.487) |
|  | Lower class | 1.986 (0.621) * | 2.604 (0.812) ** |
| Constant |  | 0.774 (0.167) | 0.792 (0.180) |

** p-value < .05, ** p-value < .01, *** p-value < .001; RRR* Relative risk ratio

XIII. Multinomial logistic regression of the association between race and vaccine myths among Utahn adults with ‘lowest-level myth’ as the base outcome

| **Variables** | **Categories** | **Medium-level myth RRR (SE)** | **Highest-level myth RRR (SE)** |
| --- | --- | --- | --- |
| **Race** | White | (ref) | (ref) |
|  | Others | 1.706 (0.655) | 2.634 (0.992) * |
| Constant |  | 1.126 (0.146) | 1.132 (0.149) |

** p-value < .05, ** p-value < .01, *** p-value < .001; RRR* Relative risk ratio

XIV. Multinomial logistic regression of the association between Hispanic status and vaccine myths among Utahn adults with ‘lowest-level myth’ as the base outcome

| **Variables** | **Categories** | **Medium-level myth RRR (SE)** | **Highest-level myth RRR (SE)** |
| --- | --- | --- | --- |
| **Hispanic status** | Non-Hispanic | (ref) | (ref) |
|  | Hispanic | 3.285 (1.679) * | 4.215 (2.152) ** |
| Constant |  | 1.086 (0.139) | 1.126 (0.146) |

** p-value < .05, ** p-value < .01, *** p-value < .001; RRR* Relative risk ratio

XV. Multinomial logistic regression of the association between political ideology and vaccine myths among Utahn adults with ‘lowest-level myth’ as the base outcome

| **Variables** | **Categories** | **Medium-level myth RRR (SE)** | **Highest-level myth RRR (SE)** |
| --- | --- | --- | --- |
| **Political ideology** | Extremely liberal | (ref) | (ref) |
|  | Fairly/somewhat liberal | 0.644 (0.298) | 0.956 (0.508) |
|  | Don't lean to either side | 1.944 (0.886) | 3.519 (1.745) * |
|  | Fairly/somewhat conservative | 1.306 (0.587) | 2.899 (1.426) * |
|  | Extremely conservative | 2.040 (1.104) | 3.941 (2.295) * |
| Constant |  | 0.908 (0.355) | 0.522 (0.230) |

** p-value < .05, ** p-value < .01, *** p-value < .001; RRR* Relative risk ratio
